# The Wigmore Hall Medical Meeting

**Published:** 1919-02-08

**Authors:** 


### THE WIGMORE HALL MEDICAL MEETING.

### A LIVELY DISCUSSION.

A Sunday afternoon meeting of the medical profession held for the discussion of medical politics must be a decided novelty, but the day and hour seemed not unsuitable to the doctors. The attendance numbered something like 400, and there was no lack of vigour in the debate, and "incidents" were numerous. The declared business of the meeting was the institution of a new organisation in which it was claimed all the various interests of the profession would be represented, and through which the opinions and decisions of the profession would be expressed to the Government and the Legislature. With a Ministry of Health impending there is need, it was urged, for the medical profession to be in a position to speak with a united voice on various questions which concern the health of the community. Only in this way can legislation be wisely inspired and directed, and the legitimate interests of the profession protected. By what organisation the meeting was summoned did not appear, and the Edinburgh professor who was to have taken the chair was unfortunately prevented from attending by illness.

Dr. Fielding Ould, who presided, had anything but an easy task, but he came through it with good temper and a reasonable degree of firmness. He protested the good intentions and personal disinterestedness of the promoters of the meeti: nd claimed that no existing organisation gave an adequate opportunity for an expression of the views of the medical practitioner. To stand aside when questions vital to the health of the community were under discussion was not possible, and hence the profession must find an organ through which its views could be declared. He called on Dr. Greenyer to move a resolution in favour of the establishment of a body representing all the interests of the profession. In doing this Dr. Greenyer found himself compelled to examine the existing organisations, and no one of these did he regard as entirely satisfactory. His criticisms of some of them evidently trod on the toes of certain of their members, for the censures passed by the speaker excited some hostile comments in the body of the hall. It soon became evident that some lively speaking was to be anticipated. The old controversy of the Insurance Act was revived as well as some more recent controversial topics, and it may be doubted whether this line of advocacy was the most politic method of securing a unanimous decision for the proposed new organisation. But the need had to be shown, and this almost compelled a proclamation of the defects of the Richmonds already in the field. In particular, the British Medical Association was condemned as having failed the profession in the Insurance Act dispute of 1913, and as unworthy of further confidence. This brought to the platform Dr. Fothergill, who vigorously defended the Association and claimed that it had a most important voice in the counsels of the Government. He regarded it as absurd that a new body should be formed when legislation was actually impending and when all the questions arising from this legislation had been fully discussed between the largest of the professional organisations and the representatives of the Government. Dr. Brackenbury also defended the B.M.A., and urged that the right policy was for the critics to come inside the Association and try to improve it rather than to stand outside and throw stones. This, too, was the view of Mr. G. B. Turner, who claimed that the democratic constitution of the Association gave an assurance that the voice of the majority must prevail, and hence no need for a new organisation existed; he urged practitioners to join the Association and to determine the policy which they desired to put into operation. The argument for the Association, however, evidently did not touch a large section of the meeting, and the applause was mainly for those who argued that the Association had been found wanting. Among the supporters of the proposed new organisation was Sir Wm. Watson Cheyne, M.P., who, while he admitted that the Association was sometimes parochial in its views, yet considered that some of the censures passed on it were unfair, and later Dr. Squire Sprigge, editor of the *Lancet* dissociated himself from the terms of these hostile criticisms. After some further speaking the motion was put to the vote and was declared carried by a majority of 182 to 93.

Now came the necessity to create some executive body to call the new organisation into existence. All that those who had summoned the meeting had to say on this point was to move that a "Provisional Committee" be formed, and the proposer and seconder of this motion had no further guidance to give on the matter. Thereupon Dr. Squire Sprigge, in a conciliatory and carefully reasoned speech, proposed that the task of organisation should be committed to the "Medical Parliamentary Committee" which, it may be remembered, was formed some months ago on the occasion of a meeting of the profession in the Steinway Hall. The speaker dwelt on the importance of the element of time in the matter and urged the suitability of the Parliamentary Committee for the work to be performed. In this he was seconded by Dr. Arthur Latham, one of the Honorary Secretaries of the Committee. On the face of it the proposal seemed a very harmless one, but it was soon the occasion of the most lively controversy. Dr. Hawthorne rose to oppose it, and his criticisms evidently engaged the sympathy of many in the audience. He thought it a most extraordinary experience that having resolved on a new departure the meeting should immediately be asked to fall back on an already existing institution. He fully recognised the position and good intentions of some members of the Parliamentary Committee, but in its warrant and capacity to speak for the profession he had

**The Wigmore Hall Medical Meeting**—(*continued*).

no confidence. It was formed for a particular and limited purpose which it had not yet achieved, and he urged the meeting to refuse to enlarge its powers in this roundabout fashion. Other speakers followed on similar lines, and in spite of interruptions and protests the meeting refused to hand over its policy to the Medical Parliamentary Committee, and the amendment was rejected by a large majority. There remained the necessity of finding a substitute, and even now the tepid proposal from the platform did not escape challenge. Dr. Angus appeared with a further amendment, namely, "That no committee or organisation of medical men is capable of effectively representing the interests of the profession unless it is a Registered Trade Union." This proposition was moved in a highly effective and eloquent speech, many passages of which were greeted with loud and prolonged applause. It was seconded by Dr. Newell, a member of the Medico-Political Union, and earlier in the afternoon a passionate speech by Dr. Guest, a Labour candidate at the recent election, had forcibly advocated the adoption of trade union methods, including, if need be, the weapon of the strike. To judge from the volume of the applause the majority of the meeting were in full sympathy with this policy, and the more timid counsels of Dr. Chapple (formerly M.P. for Stirlingshire) and Dr. A. Morrison awoke but little response. Yet when the vote was taken the chairman declared the amendment lost by seventy-three votes to seventy-one. The scene that followed was hardly creditable to the meeting. Exasperated by what they claimed as a miscount the trades union partisans demanded a new vote, and they continued loudly to voice their demand even when the chairman explained that a number of the audience after voting had left the meeting. To produce calm and to proceed with the business was impossible in face of the general disturbance, and ultimately the chairman left the chair and declared the meeting adjourned. He did not suggest any date for its renewal, and the probability is that no further attempt will be made.

A general resolution in favour of a new representative body was adopted, but the means of putting this into action remains unachieved. A large section of the meeting expressed its distrust of the British Medical Association, and with almost equal emphasis the Medical Parliamentary Committee was pushed aside. Then in searching for an alternative instrument there arose the controversy as to trades union methods in the profession, and the minority, displeased with the official count of votes, prevented all further business and the meeting broke up in confusion. What will the Professor in "the grey metropolis of the North" think of it all? And still more, what will the public think?

